# 

**Published:** 1884-01

**Authors:** 


ESTABLISHED IN 1855.

OLD SERIES.
Vol. XXI—No. 10.

JANUARY, 1884.

NEW SERIES

Vol. III—No. 4

### ATLANTA

### MEDICAL REGISTER.

*(ATLANTA MEDICAL AND SURGICAL JOURNAL.)*

EDITED BY

J. H. LOGAN, A.M., M.D.

H. H. DICKSON, PUBLISHER. TERMS, $1.50 A YEAR IN ADVANCE

ATLANTA, GEORGIA:

*H. H. DICKSON, BOOK AND JOB PRINTER AND BOOKBINDER.*

1884.

